# Supplementary material for: Cap-adjacent 2`-O-ribose methylation of RNA in C. elegans is required for postembryonic growth and germline development in the presence of the decapping exonuclease EOL-1
Source: bioRxiv. 2025 May 12:2025.03.10.638824. Preprint. [Version 2] doi: 10.1101/2025.03.10.638824 (PMC12247656; doi:10.1101/2025.03.10.638824)
Supplement: 1 [file NIHPP2025.03.10.638824V2-supplement-1.pdf]

bioRxiv preprint doi: https://doi.org/10.1101/2025.03.10.638824; this version posted May 12, 2025. The copyright holder for this preprint (which was not certified by peer review) is the author/funder, who has granted bioRxiv a license to display the preprint in perpetuity. It is made available under aCC-BY 4.0 International license.

SUPPLEMENTARY FIGURES

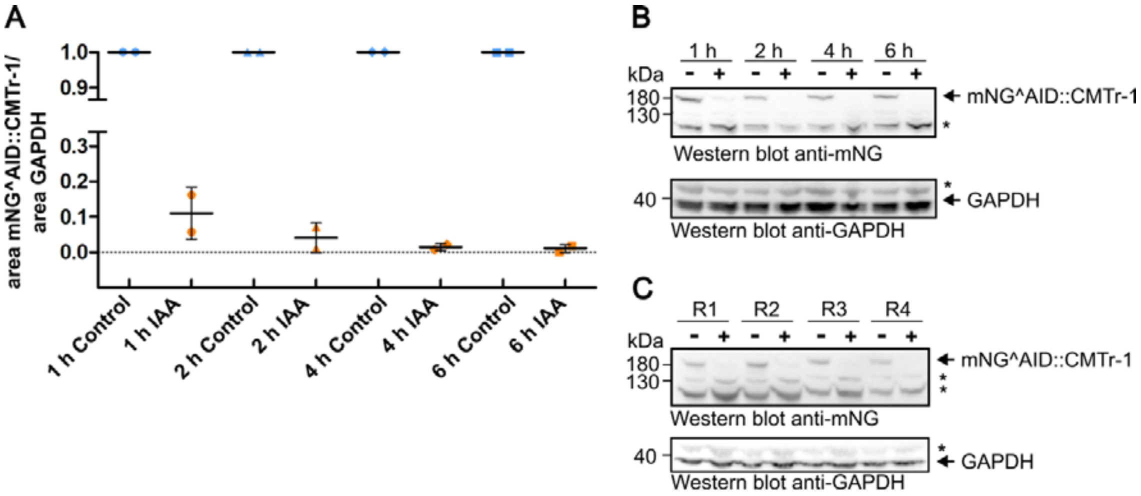

Supplementary Figure 1. 5-Ph-IAA depletion of mNG^AID::CMTR-1. (A) PE1176 animals were treated with 5-Ph-IAA or control-treated and subjected to Western blotting at the indicated time points. Proteins were detected using anti-NeonGreen and anti-GAPDH antibodies. Protein levels of mNG^AID::CMTR-1 were standardised relative to GADPH, with the controls set to 1. Data represents 2 biological replicates. (B) Representative Western blot for the data shown in A. (C) Western blot of mNG^AID::CMTR-1 depletion of replicates (R1-4) used to generate RNA-Seq data (see Figure 6). In B and C, "+" indicates 5-Ph-IAA treated samples and "-" control samples, respectively.

bioRxiv preprint doi: https://doi.org/10.1101/2025.03.10.638824; this version posted May 12, 2025. The copyright holder for this preprint (which was not certified by peer review) is the author/funder, who has granted bioRxiv a license to display the preprint in perpetuity. It is made available under aCC-BY 4.0 International license.

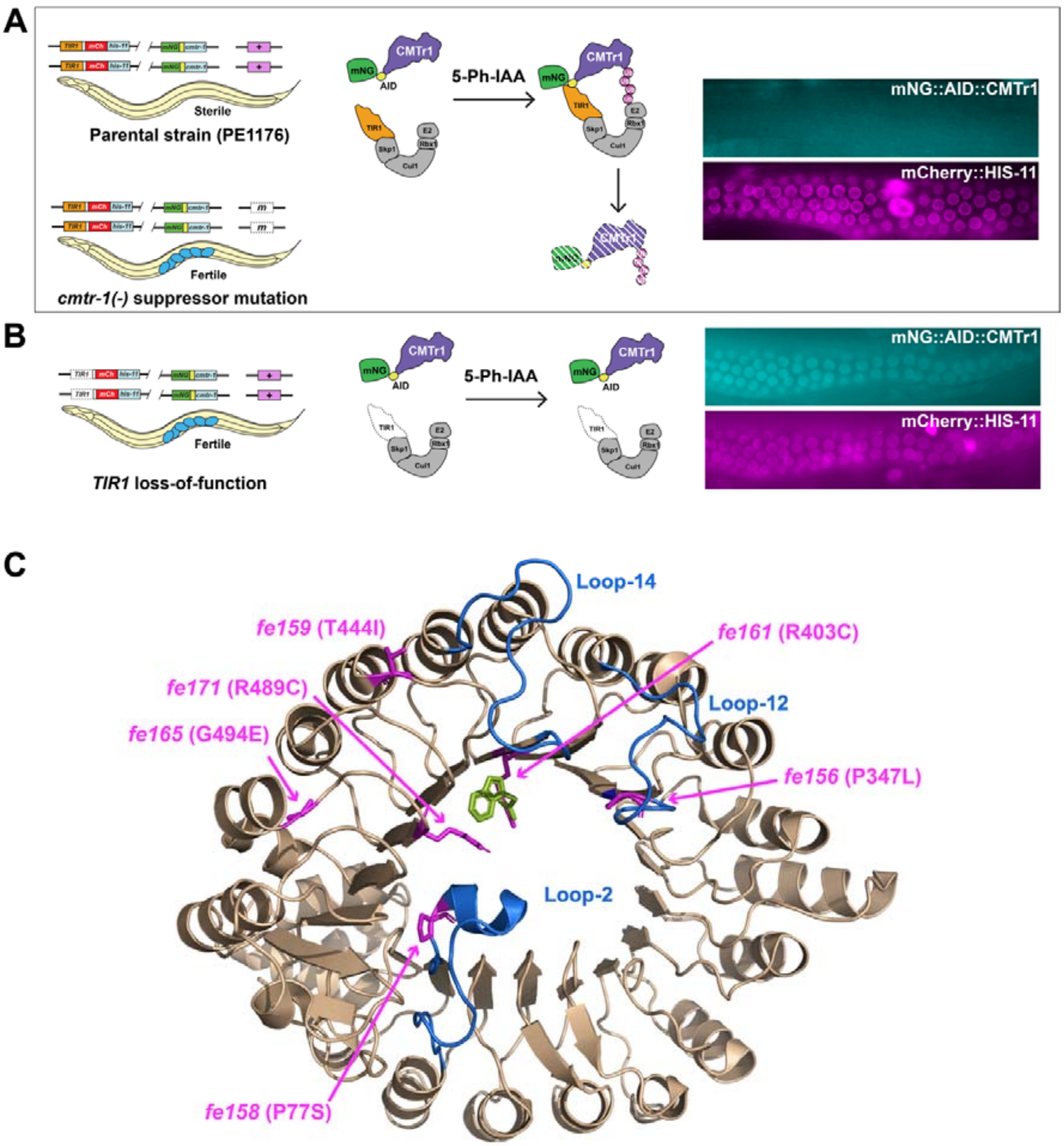

Supplementary Figure 2. Secondary screen to distinguish suppressors epistatic to *cmtr-1(-)* from *TIR1* loss-of-function mutants. (A) The starting strain for the suppressor mutagenesis screen (PE1176) is sterile, while strains carrying suppressor mutations (m, unshaded, dashed box) that are epistatic to *cmtr-1(-)* restore fertility (blue ovals indicate eggs inside gravid hermaphrodites). (B) We predicted that TIR1 loss-of-function mutants (unshaded, dashed box) would also restore fertility to *cmtr-1(-)* animals, since these would prevent depletion of mNG^AID::CMTR-1. To distinguish between these two suppressor classes, we conducted a secondary screen based on mNeonGreen fluorescence. This was absent in PE1176 and epistatic suppressor strains (panels show distal gonad arms of adult hermaphrodites; nuclear mCherry::HIS-11 fluorescence was unaffected and serves to locate

bioRxiv preprint doi: https://doi.org/10.1101/2025.03.10.638824; this version posted May 12, 2025. The copyright holder for this preprint (which was not certified by peer review) is the author/funder, who has granted bioRxiv a license to display the preprint in perpetuity. It is made available under aCC-BY 4.0 International license.

the germline nuclei), but was present in *TIR1* loss-of-function mutants. (C) Location of selected *TIR1* loss-of-function mutations identified from secondary screening (magenta). View of the TIR1-LRR domain showing the auxin and substrate-binding pocket (https://doi.org/10.2210/pdb2P1Q/pdb; (Tan et al., 2007)). Auxin is shown in green, the three extended loops key for the formation of the pocket are shown in blue.

bioRxiv preprint doi: https://doi.org/10.1101/2025.03.10.638824; this version posted May 12, 2025. The copyright holder for this preprint (which was not certified by peer review) is the author/funder, who has granted bioRxiv a license to display the preprint in perpetuity. It is made available under aCC-BY 4.0 International license.

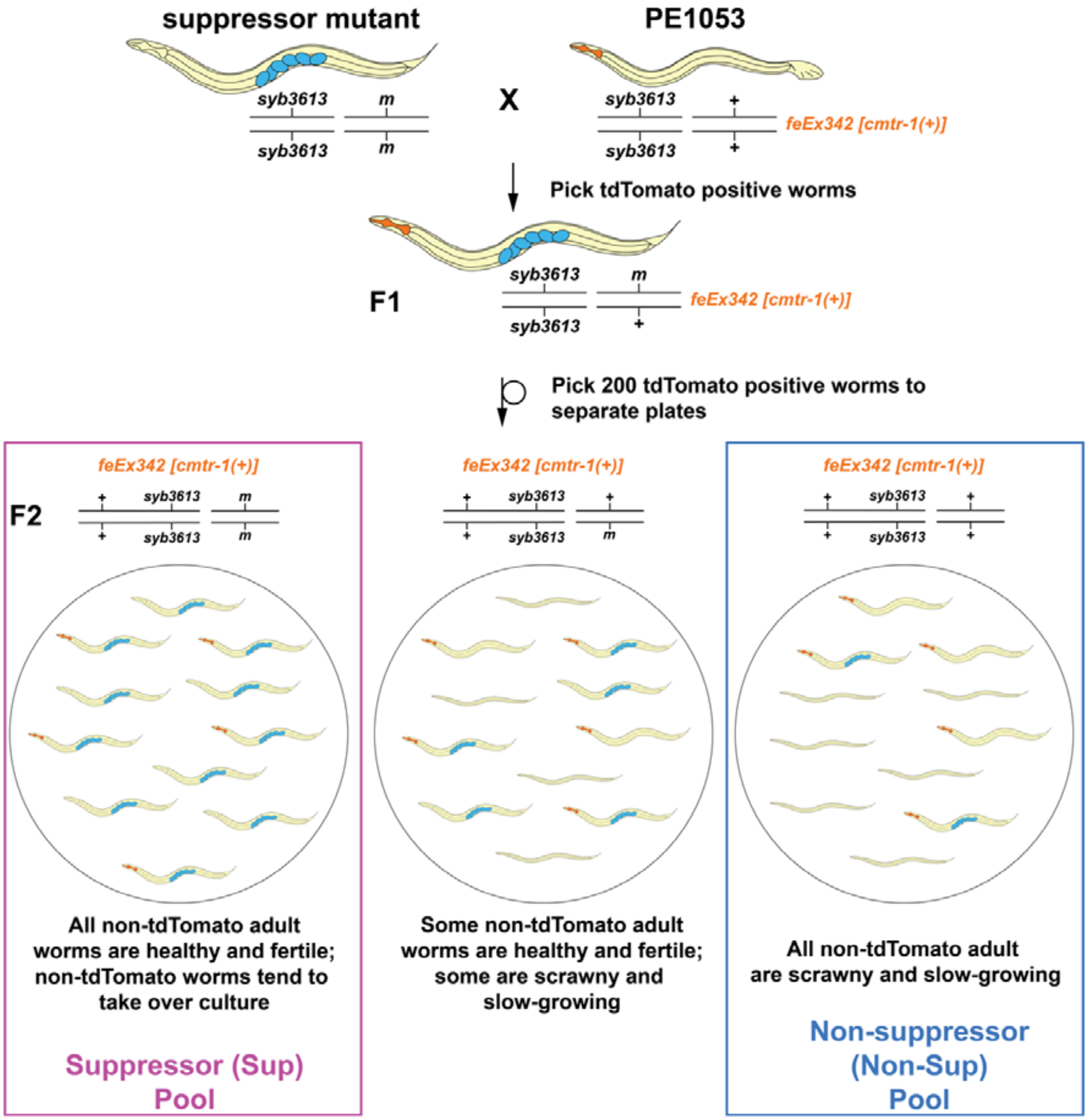

Figure 3. Strategy used to isolate strains for sibling-subtraction/whole genome sequencing mapping. Suppressor strains were crossed to the transgenic rescue line, PE1053, which is homozygous for *cmtr-1(syb3613)* but rescued by the *feEx342 cmtr-1(+)* transgene. The Sup and Non-Sup lines were established from single F2 animals on the basis that F2 suppressor homozygotes were not dependent on the *feEx342 cmtr-1(+)* transgene, while the broods of non-suppressor homozygotes resembled those of the PE1053 grandparent strain. Note, *feEx342* shows relatively poor rescue of the *cmtr-1* loss-of-function phenotype, so some transgenic animals are sterile. Transgenic animals were recognised on the basis of the *myo-2p::tdTomato* expression in the pharynx, which is also carried on the *feEx342*

bioRxiv preprint doi: https://doi.org/10.1101/2025.03.10.638824; this version posted May 12, 2025. The copyright holder for this preprint (which was not certified by peer review) is the author/funder, who has granted bioRxiv a license to display the preprint in perpetuity. It is made available under aCC-BY 4.0 International license.

extrachromosomal transgenic array. Blue ovals indicate eggs inside gravid hermaphrodites.
